# Thyroid hormone-regulated gene expression in juvenile mouse liver: identification of thyroid response elements using microarray profiling and *in silico *analyses

**DOI:** 10.1186/1471-2164-12-634

**Published:** 2011-12-29

**Authors:** Martin A Paquette, Hongyan Dong, Rémi Gagné, Andrew Williams, Morie Malowany, Mike G Wade, Carole L Yauk

**Affiliations:** 1 Environmental Health Sciences and Research Bureau, Healthy Environments and Consumer Safety Branch, Health Canada, 50 Colombine Driveway, Ottawa, Ontario, K1A 0K9, Canada; 2 Department of Biology, Carleton University, 1125 Colonel By Drive, Ottawa, Ontario, K1S 5B6, Canada

## Abstract

**Background:**

Disruption of thyroid hormone signalling can alter growth, development and energy metabolism. Thyroid hormones exert their effects through interactions with thyroid receptors that directly bind thyroid response elements and can alter transcriptional activity of target genes. The effects of short-term thyroid hormone perturbation on hepatic mRNA transcription in juvenile mice were evaluated, with the goal of identifying genes containing active thyroid response elements. Thyroid hormone disruption was induced from postnatal day 12 to 15 by adding goitrogens to dams' drinking water (hypothyroid). A subgroup of thyroid hormone-disrupted pups received intraperitoneal injections of replacement thyroid hormones four hours prior to sacrifice (replacement). An additional group received only thyroid hormones four hours prior to sacrifice (hyperthyroid). Hepatic mRNA was extracted and hybridized to Agilent mouse microarrays.

**Results:**

Transcriptional profiling enabled the identification of 28 genes that appeared to be under direct thyroid hormone-regulation. The regulatory regions of the genome adjacent to these genes were examined for half-site sequences that resemble known thyroid response elements. A bioinformatics search identified 33 thyroid response elements in the promoter regions of 13 different genes thought to be directly regulated by thyroid hormones. Thyroid response elements found in the promoter regions of Tor1a, 2310003H01Rik, Hect3d and Slc25a45 were further validated by confirming that the thyroid receptor is associated with these sequences *in vivo *and that it can bind directly to these sequences *in vitro*. Three different arrangements of thyroid response elements were identified. Some of these thyroid response elements were located far up-stream (> 7 kb) of the transcription start site of the regulated gene.

**Conclusions:**

Transcriptional profiling of thyroid hormone disrupted animals coupled with a novel bioinformatics search revealed new thyroid response elements associated with genes previously unknown to be responsive to thyroid hormone. The work provides insight into thyroid response element sequence motif characteristics.

## Background

The thyroid participates in the regulation of basic physiological processes by producing thyroid hormones (THs), which include thyroxine (T4) and triiodothyronine (T3). THs exert their effects on growth, development and metabolism of practically every cell and organ [1]. Their primary effect is the transcriptional regulation of target genes. This occurs when THs interact with TH receptors (TRs). Similar to other nuclear receptors, TR contains a DNA-binding domain that is capable of interacting with specific DNA sequences known as thyroid response elements (TREs). Typically, TREs are composed of two or more hexamer half-site sequences arranged in tandem array. TRs have the ability to bind to various imperfect TRE half-sites. The number of half-sites, the spacing between half-sites and their orientation are all features that can vary between TREs [2]. TRs can bind to TREs as monomers or homodimers, although it is thought that TRs interact as heterodimers with the retinoid × receptor (RXR) the majority of the time [3,4].

Disruption of TH physiology during critically sensitive periods in development can lead to adverse outcomes. Studies have shown that severe TH insufficiencies lead to somatic and brain growth retardation [5]. Less severe and/or transient hypothyroidism has also been shown to cause adverse structural and functional effects [6,7]. In humans, subclinical hypothyroidism during fetal development has been shown to result in reduced cognitive function [8].

Exposures to a broad array of substances, including both natural and synthetic chemicals, have been shown to alter TH physiology (see [9,10] for review). Environmental contaminants such as perchlorates [11], polychlorinated biphenyls [12], bisphenol A [13], polybrominated diphenyl ethers [14], triclosan [15] and nitrate [16] have all been shown to have a negative effect on TH function, ranging from reducing circulating THs to altering TH-signalling.

Much effort has been expended to identify direct transcriptional targets of TH [17-19]. Identification of the thyroid regulated transcriptome will provide insights into the molecular impact of THs in directing tissue function/development. We have previously characterized TH-regulated global gene expression in the livers of juvenile mice [18]. This study evaluated transcriptional effects in animals in which TH production had been severely depressed through a relatively long-term treatment with a high concentration of an antithyroid drug (6-propyl thiouracil (PTU)). This model proved inefficient for the identification of genes directly regulated by TH as the lengthy period of treatment with antithyroid substance prevented a clear interpretation of the mode of action for altered genes. It was unclear if altered gene expression was caused by direct TH action, or due to downstream effects in the liver resulting from an altered developmental trajectory.

There is overwhelming evidence that environmental contaminants can act as endocrine disrupters with possible negative consequences for human health. However, major needs in the field include the identification of key initiating events leading to impaired development and tissue function. In the current study, we apply a well-validated animal model and robust microarray analysis to investigate hepatic transcriptional response to transient hyper- and hypothyroidism. The transient treatment time in combination with conditions of both hyper- and hypothyroidism provide a more efficient approach to identify direct hepatic targets of THs during liver development. In addition to identifying key genes that are directly controlled by THs, the work sheds light on the TRE sequences that direct TR binding. To identify TH targets, livers were collected from juvenile mice (postnatal day (PND) 15) rendered transiently hypo- or hyperthyroid. This developmental period corresponds to a dramatic increase in circulating THs. In PND 15 mice, circulating T4 is higher than at any other age [20] suggesting that chemically-induced disruption of TH levels should cause a marked response in transcription. Using DNA microarrays, we identify genes that are differentially expressed between control and TH-modulated animals, and provide supporting evidence for the presence of TREs in the regulatory regions of several of these genes. Collectively, the results provide an important addition to general knowledge of the genes that are directly regulated by THs and are useful for defining an improved TRE consensus sequence.

## Methods

### Animals and Exposures

All animal care and handling was in accordance with Canadian Council for Animal Care Guidelines and was reviewed by the Health Canada Animal Care Committee prior to commencement of the study.

C57BL/6 mice were purchased from Charles River (St. Constant, QC, Canada) and were housed in hanging polycarbonate cages under a 12:12 hr light-dark cycle at 23°C. Animals were provided with shelters, nesting material, food *ad libitum *(Purina rodent chow 5010; Ralston-Purina, St. Louis, MO, USA) and drinking water containing 1% (wt/vol) sucrose (*ad libitum)*. Mice were acclimated for 10 days. Breeding was accomplished by transferring two sexually mature female mice (eight weeks post natal) into the home cage of a sexually mature (10 weeks post natal) male. After four nights of co-housing, each female was transferred to a separate cage. Dams were allowed to litter naturally and pup numbers were not adjusted. To produce hypothyroid (hypo) pups, dams were provided with drinking water that contained methimizole (MMI, 0.05% wt/vol) and perchlorate (1% wt/vol) for three days (PND 12 to 15). To produce hyperthyroid (hyper) pups, intraperitoneal injections (i.p.) of THs (50 μg of T4 + 5 μg of T3 per 100 g body weight) were administered to pups on PND 15, four hours before decapitation and tissue collection. For the hypothyroid/replacement group (hypo+); dams were provided with drinking water that contained MMI (0.05% wt/vol) and perchlorate (1% wt/vol) for three days (PND 12 to 15). Pups then received *i.p*. injections of THs (20 μg of T4 + 2 μg of T3 per 100 g body weight) on PND 15, four hours before decapitation and tissue collection. The control and hypo groups received saline *i.p*. injections four hours before decapitation and tissue collection.

### Tissue Collection, RNA Extraction and Purification

On PND 15, pups were sacrificed by decapitation. Serum prepared from trunk blood using serum separator tubes (BD Biosciences, Mississauga, ON, Canada) was used for T4 analyses by radioimmunoassay (RIA) kits (MP Biomedicals, Medicorp, Montreal, QC, Canada) as per the manufacturer's instructions. The hypo + group was subdivided into two sub groups - hereafter called hypo+ and hypo++ - based on measured serum T4 levels. Animals allotted to the hypo++ group had serum T4 levels that approximated those seen in the hyperthyroid group while those that remained as hypo+ had T4 levels near or slightly in excess of control levels.

Pup livers were rapidly dissected and flash frozen in liquid nitrogen. Total RNA was extracted from liver samples with TRIzol reagent (Invitrogen, Burlington, ON, Canada) followed by RNeasy Mini Kit (Qiagen, Missisauga, ON, Canada) clean-up according to the manufacturer's instructions. RNA quality assessment was determined by Nanodrop (Thermo Scientific, Billerica, MA, USA) and Agilent 2100 Bioanalyzer and RNA 6000 NanoLab Chip Kit (Agilent Technologies, Mississauga, ON, Canada). All samples had 260/280 ratios over 2.1, and RNA integrity numbers over 9.0.

### Microarray Hybridization

Hepatic gene expression was assessed by hybridizing samples to Agilent 4 × 44 k Whole Genome Microarrays (G4122F) using a reference design (details below). Briefly, 200 ng of total RNA from the liver was used to synthesize double-stranded cDNA and cyanine labelled cRNA. Experimental samples were labelled with Cyanine 5-CTP and reference RNA (universal mouse RNA; Agilent Technologies) with Cyanine 3-CTP (Perkin-Elmer Life Sciences, Woodbridge, ON, Canada) according to the manufacturer's instructions (Agilent Linear Amplification, Agilent Technologies). Cyanine labelled cRNA targets were *in vitro *transcribed using T7 RNA polymerase and purified using RNeasy Mini Kit (Qiagen). Experimental and reference samples (825 ng each) were hybridized to arrays at 60°C for 17 hours. Slides were washed and scanned on an Agilent Microarray Scanner (G2565CA). The data were acquired using Agilent Feature Extraction software version 10.1.1.1. Prior to statistical analysis, the scans were inspected using the Agilent Quality Control report as well as an internal quality control metric including spike-in RNA controls.

### Experimental Design and Statistical Analysis of Microarray Data

The Agilent 4 × 44 k Whole Genome Microarray features four sub-arrays per slide. A reference design [21,22] with sub-arrays as blocks of size two (each block containing the corresponding reference: Cy3 = green, and sample: Cy5 = red channels) was used to analyze the median signal intensities of the two-color microarray data. The experiment included the main effects of treatment groups (four conditions: hyper, hypo, hypo+, and one control) plus the sub-array as a block term. Five biological replicates per condition were used for three of the four groups, while four replicates were used for the remaining hypo+ condition (due to a shortage of animals with suitable serum T4 levels in that group), yielding a total of 19 microarrays. An identical experimental structure was followed for each gender. Separate statistical analyses were carried out for the male and female samples, respectively, to eliminate any correlation effect due to the dam as samples within genders were independent.

All pre-processing of the data was conducted using R. The data were normalized using loess normalization [23] in the R library "MAANOVA". Background fluorescence was measured using the (-)3xSLv1 negative control probes; probes with median signal intensities less than the trimmed mean (trim = 5%) plus three trimmed standard deviations of the (-)3xSLv1 probes were flagged as absent (within the background signal). Ratio intensity plots (also known as MA plots) were constructed for the raw and normalized data for each array. Boxplots and hierarchical clustering using average linkage were generated to identify outlier arrays [24]. Two outlier microarrays were removed from each data set (two microarrays from male samples and two from female samples) based on separate hierarchical clustering of each dataset into sample = Cy5 and reference = Cy3 groups. As a result, the final analysis was based on 17 microarrays for females, and 17 for males.

Genes that were up- and down-regulated in any of the three treatment groups (hyper, hypo, and hypo+) relative to control were identified using the R library "MAANOVA" [25]. The required ANOVA model was fitted to include the main effects of dose plus the sub-array as a block term. The Fs statistic [26], a shrinkage estimator, was used for the gene-specific variance components, and the associated p-values for all the statistical tests were estimated using the permutation method (30,000 permutations with residual shuffling). These p-values were then adjusted for multiple comparisons using the false discovery rate (FDR) approach [27]. All data are available through the Gene Expression Omnibus (GEO) website, accession number: GSE21307.

The least squares mean [28,29], a function of the model parameters, was used to estimate the fold-change for each pairwise comparison of interest (control versus each of the three treatment groups: hyper, hypo, and hypo+.)

### Bioinformatics

Promoter regions (-8 kb to +2 kb relative to the transcription start site (TSS)) of genes whose expression profile suggested direct regulation by THs were downloaded from the UCSC Genome Browser (mm9 assembly). Using a list of validated mouse TREs gathered from the literature, a position weight matrix (PWM) was developed to score the information content (bits) for position one to six of TRE half-sites. In this way, each TRE half-site was assigned a 'score' against the PWM. The score for each TRE half-site was obtained iteratively by leaving the subject out of the PWM construction. Previous bootstrapping analysis of the validated TREs in mice revealed a difference (p < 0.001) between the distribution of scores for the two half-site scores. Cross-validation of the TREs from the literature was carried out with each half-site analyzed separately. This analysis revealed that a low threshold score (i.e., ≥ 3.76 bits) for one half-site and a high threshold score (i.e., ≥ 6 bits) for the second half-site (regardless of half-site order) allowed minimization of type I and type II errors (manuscript in preparation). We therefore used the cut-off scores identified in this cross-validation exercise and scanned the promoter regions for putative TREs. The scan searched for three different types of TREs: direct repeats with a four nucleotide spacer (DR4); inverted repeats with no spacer (IR0); and everted repeats with a spacer of six nucleotides. (ER6).

Orthologs in humans and in rats were matched using BioMart http://www.biomart.org for the genes that were identified as having potential TREs in mice. Promoter regions (defined as -8 to +2 kb of the TSS) for these orthologous genes were obtained. A local alignment using a sliding window principle was used to align the TREs identified in mice to the promoter regions of the corresponding orthologous genes in rats and humans. A modified substitution matrix favouring conservation of the guanine in position two and three of the TRE hexamer half-sites was used, since these two sites appear to be highly retained. Three additional features were used to filter identified TREs: i) a maximum mismatch of two when comparing mouse to rat, and mouse to human, ii) a maximum distance of 2 kb between TREs when comparing mouse to rat and mouse to human, and iii) a minimum score determined by the PWM scan.

### Gene Ontology Analysis and Principal Component Analysis

Genes with FDR corrected p-values smaller than 0.05 in at least one experimental condition were used for principal component analysis (PCA) and pathway/ontology analysis. PCA was carried out using mean centering and scaling on data from genes that were significant in at least one treatment group using Genespring GX 7.3.1 (Agilent Technologies). Mean fold-change, averaged across all replicates per sex per treatment, was subjected to functional enrichment and Gene Ontology (GO) analysis using Ingenuity Pathway Analysis (Ingenuity Systems Inc., Redwood, CA, USA).

### Real-Time Quantitative PCR Analysis

Total RNA was reverse transcribed into cDNA using SuperScriptIII (Invitrogen) as per the manufacturer's instructions. iQ SYBR Green Supermix (BioRad Laboratories, Mississauga, ON, Canada) was used with a reaction volume of 50 μL. All primers were designed using Beacon Designer 7 (Premier BioSoft International, Palo Alto, CA, USA). Hprt was used as the reference gene. Microarray analysis and RT-qPCR analysis confirmed that Hprt expression was stable across all groups. Reactions were carried out in 96-well plates. PCR were done in duplicate and each plate contained all samples for the gene of interest and reference gene for one sex. Analysis was carried out using a CFX96 Real-Time PCR Detection Systems (BioRad Laboratories). Melting curves were performed for each reaction to ensure primer specificity. Raw data were up-loaded into Relative Expression Software Tool 2009 (REST 2009; Qiagen). where fold-changes and p-values were calculated.

### ChIP-PCR

Livers from the male euthyroid control group were used for the ChIP using the EZ ChIP kit (Millipore Corporation, Toronto, ON, Canada), according to the manufacturer's instructions. Briefly, a small piece of liver was homogenized with a hand-held homogenizer in 250 μL PBS containing broad-spectrum protease inhibitors, and was then cross-linked with 1% formaldehyde. Cross-linking was stopped with glycine and nuclei were collected by adding lysis buffer. To ensure that DNA fragments ranged from 200 to 600 bp, the nuclear solution was sonicated in an ice bath with 30 second bursts at 28% amplitude. Fifteen bursts were completed, each separated by a 60 second period. DNA fragment size was verified by agarose gel electrophoresis. Six percent (about 100 μL) of the sonicated solution was stored at -20°C as total input (TI), while the remainder was incubated with anti-TRβ polyclonal antibody (PA1-213, clone TRβ-62, Affinity BioReagents, Golden, CO, USA) overnight with agitation at 4°C. Antibody-bound chromatin was precipitated with Protein G conjugated agarose beads, washed with gradient stringent buffers, and eluted with elution buffer as per the manufacturer's instructions. Both the eluted solution and the stored TI were incubated at 65°C overnight to reverse cross-links. Immunoprecipitated (IP) DNA and TI DNA were purified by treatment with RNase, proteinase K and multiple phenol:chloroform:isoamyl alcohol (25:24:1) extractions. Equivalent amounts of IP DNA and TI DNA were amplified in parallel, using a random primer method with GenomePlex Complete Whole Genome Amplification Kit (Sigma-Aldrich, Oakville, ON, Canada). Amplified DNA was then purified using GenElute PCR Clean-Up kit (Sigma-Aldrich).

Primers targeting the TRE identified by bioinformatics analysis were designed using Beacon Designer 7. PCRs were performed using Expand High Fidelity PCR System (Roche, Laval, QC, Canada). β-actin (NM_007393.3) was used as a negative control and Mlxipl (NM_021455.3) was used as a positive control [30]. Gel analyses and band quantifications were carried out using GeneTools (Syngene, Frederick, MD, USA). Binding ratios were calculated by dividing the ratio of IP/TI from the gene of interest by the ratio of IP/TI from the reference gene β-actin. Mean enrichment, standard deviations, and p-values were calculated using log ratios and were then back-transformed.

### Electrophoretic Mobility Shift Assays

Probes were designed against the specific genomic regions of the newly identified TREs. Probe size ranged between 26 and 30 bp. The probe contained the putative TRE sequence plus six to seven nucleotides on each side. Oligonucleotides were labelled using Biotin 3' End DNA Labeling Kit (Thermo Scientific) as per the manufacturer's instructions and were then annealed. Binding reaction and detection of complexed biotin-labeled DNA-protein was accomplished using the LightShift Chemiluminescent EMSA Kit (Thermo Scientific) as per the manufacturer's instructions. Binding reactions were carried out in 20 μL volumes and had final concentrations of 2.5% glycerol, 5 mN MgCl_2_, 50 ng/μL Poly (dI•dC) and 0.05% NP-40. One μg of chicken TRα (Santa Cruz Biotechnology Inc., Santa Cruz, CA, USA) was used per reaction. An amino acid alignment of the TRα DNA-binding domains from *Mus musculus *and *Gallus gallus *showed an approximate 98% sequence homology. Amino acids involved in direct base contacts [31] were specifically examined and were not found to be substituted between species. We have previously found that TRα and TRβ interact with TREs in a very similar manner [18]. Amino acid alignment of DNA-binding domains showed an approximate 86% sequence homology between the TR isoforms. Amino acids involved in direct base contacts [31] were specifically examined and showed 100% conservation between the two isoforms.

Biotin end-labelled target DNA was diluted 1:4 and 2 μL were used per reaction. Unlabeled probes were used at a 200-fold molar excess compared to labelled probe. For the supershift, 2 μg of antibody TRα/β (Santa Cruz Biotechnology Inc.) or normal mouse IgG (Santa Cruz Biotechnology Inc.) were used per reaction. Samples were run on a 5% polyacrylamide gel, transferred onto Biodyne Precut A Nylon Membranes (Thermo Scientific) by electrophoretic transfer and UV-cross-linked. Membranes were scanned using ChemiDoc XRS+ (BioRad Laboratories) and images analysed using Image Lab version 2.0.1 (BioRad Laboratories)

## Results

### Validation of the MMI/sodium Perchlorate Mouse Model

**Figure 1:**
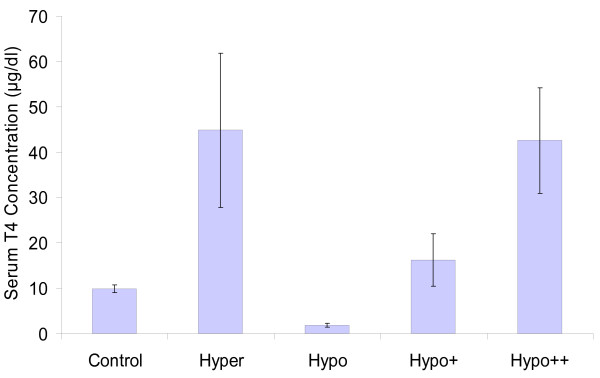
**PND 15 pup serum T4 levels after short-term TH perturbation**. Means are presented as well as ± standard deviations. n = 10, 5 from each sex, for control, hyper and hypo; n = 8, 4 from each sex for hypo+; n = 5, males only for hypo++. All treatment groups when compared to controls were significantly different; p ≤ 0.05, determined by student t-test.

Serum T4 levels in all PND 15 pups were measured (Figure 1). A significant increase was observed in the hyper pup group compared to control levels, whereas a significant decrease was observed in the hypo pup group compared to control levels. The hypo+ and hypo++ pup groups also showed a significant increase in serum T4 compared to control. The hypo++ pup group received the same doses of THs in the *i.p*. injection as the hypo+, but registered higher levels of circulating T4 (levels similar to the hyperthyroid pup group). Levels of T4 in hyperthyroid and hypo++ groups were not significantly different. Part of this data has already been reported [32].

### Genes Significantly Altered by TH Perturbations

MAANOVA analysis identified approximately 400 significantly altered genes in male and/or female pups, with a FDR-adjusted p < 0.05 in at least one of the treatment conditions (see Additional File 1 for male data and Additional File 2 for female data). Fold-changes ranged from 1.1 to 13.8, and averaged approximately 1.6. In hypo mice, 215 genes were significantly altered relative to euthyroid controls. Of these, transcription levels of 118 genes were reduced and 97 were increased in hypo livers relative to controls. In the hyperthyroid group, 204 genes were significantly altered. Of these, transcript levels of 45 genes were reduced and 159 were increased relative to controls. In the hypo+ group, 68 genes were significantly altered with 14 reduced and 54 increased. Females exhibited more significantly altered genes than males. Females had 300 significantly altered genes whereas males had 185. A comparison of these two lists revealed that there were 100 genes that were differentially expressed by at least one treatment condition in both females and males (i.e., 100 genes in common between the sexes).

**Figure 2:**
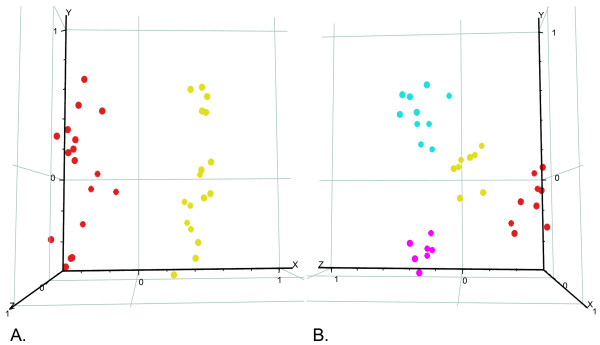
**Principal component analysis of microarray data carried out with the full set of significant genes (FDR-adjusted p ≤ 0.05) for all groups (control, hyper, hypo and hypo+)**. Data are coloured by sex (A) and by treatment group (B). In panel (A) male data points are in red and female data points are in yellow, and in panel (B) control data points are in yellow, hypothyroid data points are in red, hyperthyroid data points are in cyan and hypothyroid/replacement (hypo+) data points are in fuchsia.

Using the full set of significant genes (FDR-adjusted p < 0.05) for all groups (control, hyper, hypo and hypo+), a principal component analysis (PCA) was conducted (Figure 2). This analysis revealed that expression patterns were highly correlated within sexes and treatment groups. The PCA revealed distinct male and female clusters (Figure 2A) and four clusters were also found to separate control, hyper, hypo and hypo+ groups. Thus, the expression profiles reveal both an overall treatment as well as sex effect, with both sexes showing similarities in their response within treatment groups.

The list of all significant genes was subjected to GO and pathway analysis. The analysis revealed several pathways that were significantly altered by the treatment. The five most affected pathways are presented in Additional File 3, along with the names of the genes that were affected by TH treatment within the pathway. Many genes were found to be involved in oxidative stress response and xenobiotic metabolism/signalling. A group of genes involved in TR/RXR activation was also identified.

By comparing the response profile of genes that were significantly altered in the hyper, hypo and hypo+ groups, we identified a subset of genes that are most likely to be directly regulated by TH rather than by downstream effects. For example, a directly regulated gene would be expected to show similar responses in the hyper and hypo+ groups, and should be in the opposite direction to the hypo group. The hypo+ group is particularly informative, as these animals were sacrificed four hours after a single injection of TH, but following a more prolonged state of hypothyroidism. In addition, genes showing similar responses in hypo and hypo+ groups would be less likely to be directly regulated by TH. Genes showing a pattern suggesting direct regulation by TH are listed in Table 1.

**Table 1:** Genes identified as being directly regulated by THs.

		Hypo		Hyper		Hypo+	
		
Accession	Gene	♂	♀	♂	♀	♂	♀
NM_027980	2310003H01Rik			1.4	1.4	1.3	
AK014609	4633401B06Rik				1.4	1.6	1.7
AK044145	AK044145			2.6	2.7	2.1	1.9
NM_025404	Arl4d			2.0		2.0	
NM_177047	Auts2 *			3.1	3.0	2.5	2.5
NM_145603	Ces2			1.5	1.4		1.5
NM_134141	Ciapin1			1.4	1.4	1.3	
NM_026424.3	Coq10b				1.5	2.0	
NM_001025384.3	DXBay18		-1.7	2.2	1.7	2.6	2.3
NM_175266	Epm2aip1			1.3	1.3	1.3	
NM_008058	Fzd8			1.8	1.5	1.6	
NM_153528	Gramd1c			1.6	1.7		1.4
NM_175244	Hectd3				1.3		1.4
NM_010544	Ihh *				-1.2		-1.2
NM_008358	Il15ra				1.2		1.3
NM_178701	Lrrc8d			1.6	1.4	1.6	
NM_172821	Map3k13			1.6		1.7	
NM_027418	Mapk6			1.3	1.4		1.3
NM_027997	Serpina9 *				-1.7		-1.9
NM_008018.4	Sh3pxd2a			1.7	1.7	1.5	1.5
NM_134154	Slc25a45 *	-1.7	-1.6	1.7	1.9	1.5	1.6
NM_172463	Sned1 *	-1.6	-1.6	1.5	1.5	1.5	1.4
NM_001081103	Stim2			1.9	2.0	1.5	
NM_173038.2	Tbcel			1.3	1.3	1.3	1.4
NM_144884	Tor1a				1.2	1.3	
NM_145076	Trim24 *	-1.4	-1.6	1.6	1.4	1.5	
NM_153162	Txnrd3				1.2	1.3	
XM_001481284	Wipf3	-1.4		1.4		1.3	

Fold-changes for males and females are presented in comparison to control animals. All fold-changes have an adjusted p ≤ 0.05. Asterisks (*) denote genes that were analysed by RT-qPCR. See Additional File 1 and 2 for p-value data.

### Microarray Data Validation and Hypothyroid Replacement Analysis

Microarray data were validated using real time RT-qPCR for subsets of significantly altered genes (FDR p < 0.05). First, we investigated the responses of three genes that have previously been shown to be directly regulated by TH (Thrsp, Dio1 and Me1; Table 2). Some of these genes were only significant with RT-qPCR (e.g. Dio1 in hyper males and females was not identified as differentially expressed on the microarray; Table 2). Thus, the data produced by the microarray analysis are likely to be conservative, and include some false-negatives.

**Table 2:** Microarray and RT-qPCR results for known TH-regulated genes.

		Microarray		RT-qPCR	
		
Gene	Group	FC	p-value	FC	p-value
**Male Pups**					
Thrsp	Hypo	**-2.8**	**0.036**	**-5.0**	**0.032**
	Hyper	1.6	0.999	2.4	0.170
Dio1	Hypo	**-2.6**	**0.000**	**-4.9**	**0.015**
	Hyper	1.3	0.999	**1.5**	**0.029**
Me1	Hypo	-1.4	0.379	-1.4	0.099
	Hyper	1.3	0.632	**1.5**	**0.017**
**Female Pups**					
Thrsp	Hypo	**-2.7**	**0.000**	**-5.0**	**0.033**
	Hyper	**2.4**	**0.019**	**4.6**	**0.010**
Dio1	Hypo	**-3.2**	**0.000**	**-7.8**	**0.001**
	Hyper	1.2	0.983	**1.5**	**0.008**
Me1	Hypo	**-1.4**	**0.045**	-1.3	0.143
	Hyper	1.3	0.095	**1.7**	**0.002**

Fold-changes are presented for treated animals relative to control animals; n = 4 for all groups except for hyper, where n = 5. Fold-changes (FC) and p-values are in bold when p ≤ 0.05.

Second, we validated several genes suspected of being directly regulated by TH using RT-qPCR. For this analysis, we compared gene expression changes in the four treatment groups, as well as in a group of animals subjected to the hypo+ treatment but whose circulating T4 levels were substantially higher than those in the hypo+ animals used for microarray analysis (i.e., the hypo++ group). Here we present the results from the hypo+ and hypo++ groups when compared to control data (Table 3). The direction of fold-change was consistent with the microarray data for each of the nine genes selected for RT-qPCR validation. A significant fold-change in the hypo++ group was observed in all genes evaluated except for Tor1a and Trim24.

**Table 3:** Microarray and RT-qPCR results for the male hypo+ and male hypo++ treatment groups.

		Microarray		RT-qPCR	
		
Gene	Group	FC	p-value	FC	p-value
Slc25a45	Hypo+	**1.5**	**0.000**	1.6	0.314
	Hypo++			**2.4**	**0.000**
Hectd3	Hypo+	*1.4	**0.000**	*2.4	**0.026**
	Hypo++			**1.7**	**0.005**
H01Rik	Hypo+	**1.3**	**0.026**	**2.8**	**0.011**
	Hypo++			**1.4**	**0.008**
Tor1a	Hypo+	**1.3**	**0.026**	1.6	0.117
	Hypo++			1.1	0.501
Auts2	Hypo+	**2.5**	**0.000**	**5.3**	**0.006**
	Hypo++			**5.6**	**0.002**
Serpina9	Hypo+	*-1.9	**0.000**		
	Hypo++			**-5.0**	**0.000**
Sned1	Hypo+	**1.5**	**0.000**	**2.4**	**0.027**
	Hypo++			**2.4**	**0.041**
Trim24	Hypo+	**1.5**	**0.000**	2.9	0.398
	Hypo++			1.5	0.352
Ihh	Hypo+	*-1.2	**0.047**		
	Hypo++			**-2.0**	**0.041**

The hypo++ group was not included in the microarray analysis. The fold-changes represent treated relative to control animals; for RT-qPCR data n = 5 for each group. Fold-changes (FC) and p-values are in bold when p ≤ 0.05. Asterisks (*) denote that the data were taken from female pups due to the absence of significant data from male pups. H01Rik corresponds to 2310003H01Rik. Absence of data for RT-qPCR values were because of undetectable signal due to low expression level.

### Bioinformatics Analysis of Direct TH-Regulated Genes

A search for TREs in the 28 regulatory regions of genes suspected to be under direct TH-regulation (Table 1) identified 196 TREs in 24 different genes. When locally aligned with the rat promoter region of the corresponding orthologs, the list was reduced to 68 TREs in 20 different genes. Once this was accomplished, the remaining TREs were locally aligned with the human promoter region of the corresponding orthologs to produce the final list of 33 TREs found in 13 different genes (Table 4). The candidate TRE sequences in rats or humans were within 2 kb of the relative distance of the putative TRE identified in mice, and had two or less mismatches when compared to the mouse TRE. Thus, this final list of 33 TREs provides the most likely candidate TRE-containing promoters that may be conserved between mice, rats and humans.

**Table 4:** TREs identified using bioinformatics searches of promoter regions of genes characterized as being directly regulated by THs.

		Mouse		Rat			Human		
		
Gene	Type	**Pos**.	TRE	**Pos**.	TRE	MM	**Pos**.	TRE	MM
H01Rik^‡^	DR4	1597	**GGGTCA**CCAG**GGGCTA**	1513	**GGGTCA**CCAG**GGGCTA**	0	1709	**GGGTCA**CCAG**GGGCTG**	1
H01Rik^‡^	DR4	-5778	**AGGGCA**GCAG**AGCTGA**	-5806	**AGGGCA**GCAG**GGCTGA**	1	-7091	**AGGGGC**AGGC**AGCTGA**	1
							-4046	**AGGGAA**AGAC**AGCTCA**	2
H01Rik^‡^	DR4	281	**AGGTCA**GGCG**AGGGCA**	243	**AGGTCA**GGCG**AGGGCA**	0	1176	**TGGTCA**GGCC**TGGGCA**	2
H01Rik^‡^	IR0	-2311	AGGTGAAGCCCT*	-2421	**AGGTGAAGCCCT**	0	-3415	**CGGTGAAACCCT**	2
Arl4d	DR4	-2529	**AGGCCA**GCCA**GGGCTA**	-1594	**AGGCTA**GCCT**GGGCTA**	1	-1286	**AGGCCT**CAAA**GGGCTT**	2
							-922	**AGGCCG**ATGT**GGGCGA**	2
Ces2	DR4	-5950	**AGGCAA**AGCA**AGGTCT**	-7139	**AGGCAA**CTGC**AGGTTT**	1	-7548	**AGGCAA**AAGC**TGGGCT**	2
							-7219	**TGGCAA**TCTG**AGCTCT**	2
							-4185	**AGCTAA**GCCA**AGGTCT**	2
Fzd8	DR4	-143	**CGGTCA**CCCC**AGGAGA**	6	**CGGTCA**CCCC**AGGAGA**	0	399	**CGGTCG**GGCC**AGGCGA**	2
Hectd3	ER6	-7433	**TGGCCT**GAAGATAGGACA*	-6784	**TGGCCT**CTCCTA**TGGACA**	1	-7570	**TGGCCT**ACAACC**AGGATA**	1
							-6806	**GGGCCA**ATGCTC**AGGACA**	2
Hectd3	DR4	-3516	**AAGTCA**CCTG**AGGAGA**	-5475	**AAGTCA**CCTG**AGGAGA**	0	-2992	**AAGTCA**TTTG**GGGAAA**	2
Ihh	ER6	-5766	**TGACCT**TTATGC**AAGTCA**	-6131	**TGACCT**TTATTC**AAGTCA**	0	-4898	**CCACCT**CTGTTC**AAGTCA**	2
Serpina9	DR4	31	**AGGACA**ACAA**GGGCGA**	31	**AGGACA**ACAA**AGGCGA**	1	167	**AGGACA**GGGC**AGGAGA**	2
Slc25a45	DR4	-4642	**AGGATT**TCTAAGGCCA*	-5175	**AGGATT**TCTA**TGGCCA**	1	-6373	**AGGTTT**GCAA**TGGCCA**	2
Sned1	DR4	409	**AGGTGG**AATG**AGGACA**	317	**AGGTGG**AATG**AGGACA**	0	332	**AGGTGG**AATG**AGGACA**	0
							-864	**AGGTGG**GGGC**AGGACT**	1
Sned1	ER6	217	**TCACCC**CGAAGC**AGGACG**	125	**TCACCC**CGAAGC**AGGACG**	0	125	**TCACCC**CCAAGC**AGGACG**	0
Tbcel	DR4	15	**GGGTCA**GCAT**AGGACA**	-7	**GGGTCA**ATGC**AGGACA**	0	15	**GGGTCA**ATGC**AGGACA**	0
Tbcel	IR0	920	**AGGACAAGTCCC**	891	**AGGACAAGTCCC**	0	649	**AGGACATGTCCC**	1
							1157	**GGGACAAGCCCC**	2
							470	**GGGACCAGTCCC**	2
Tbcel	DR4	1361	**AGGCCA**GCCT**GGGCTA**	1371	**AGGCCA**GCCT**GGGCTA**	0	-230	**AGGACA**ATAG**GGGCTG**	2
Tor1a	DR4	-2364	**AGGACA**GCCAGGGCTA*	-4242	**AGGAAA**CACA**CGGCTA**	2	-1610	**AGGATA**CTCC**GGGCTC**	2
Tor1a	DR4	1648	**AGGTTA**GTCT**GGGCTA**	1638	**AGGTTA**GTCT**GGGCTG**	1	552	**CGGTTG**GCTG**GGGCTA**	2
Tor1a	DR4	-2729	**AGGACA**GCCA**GGGCTA**	-4242	**AGGAAA**CACA**CGGCTA**	2	-1610	**AGGATA**CTCC**GGGCTC**	2
Tor1a	DR4	-4783	**AGGCCA**CTTC**AGGTTG**	-6425	**AGGCCA**GAAG**AGGGTG**	1	-6272	**AGGCCA**AGGC**AGGAGG**	2
							-6628	**AGGCCA**GGCA**CGGTGG**	2
Trim24	DR4	295	**AGGACA**ATGG**AGGTGG**	-5	**AGGACA**ATGG**AGGTGG**	0	336	**AGGACA**ATGG**AGGTGG**	0
Txnrd3	IR0	372	**GGGTGATGATCT**	362	**GGGTTATGATCT**	1	1423	**GGGTGGTGATCT**	1
							1876	**GGGTGATAACCT**	2
**Previously Characterized TREs**									
Klf9^a^	DR4	-3804	**AGGTGA**AGTG**AGGTCA**	-3819	**AGGTGG**GGCG**AGGTCA**	1	-2875	**AGATTG**TCTG**AGGTTA**	4
Mbp^b^	ER6	-192	**GGACCT**CGGCTG**AGGACA**	-138	**GGACCT**CGGCCG**AGGACA**	0			

TREs were found to be conserved between mice, rats and humans (i.e., within 2 kb of the mouse TRE and with 2 or less mismatches (MM) when compared to the mouse TRE). MMs were only considered in the bolded half-sites. Positions (Pos.) of the TREs are presented in reference to the transcription start site of the associated gene. Two previously characterized TREs are presented at the bottom of the table. * TREs validated by ChIP-PCR. ^‡ ^Corresponds to 2310003H01Rik. ^a ^Characterized by [40]. ^b ^Characterized by [41,42]; bolding of half-sites and positions of TRE were corrected.

### ChIP-PCR Analysis

**Figure 3:**
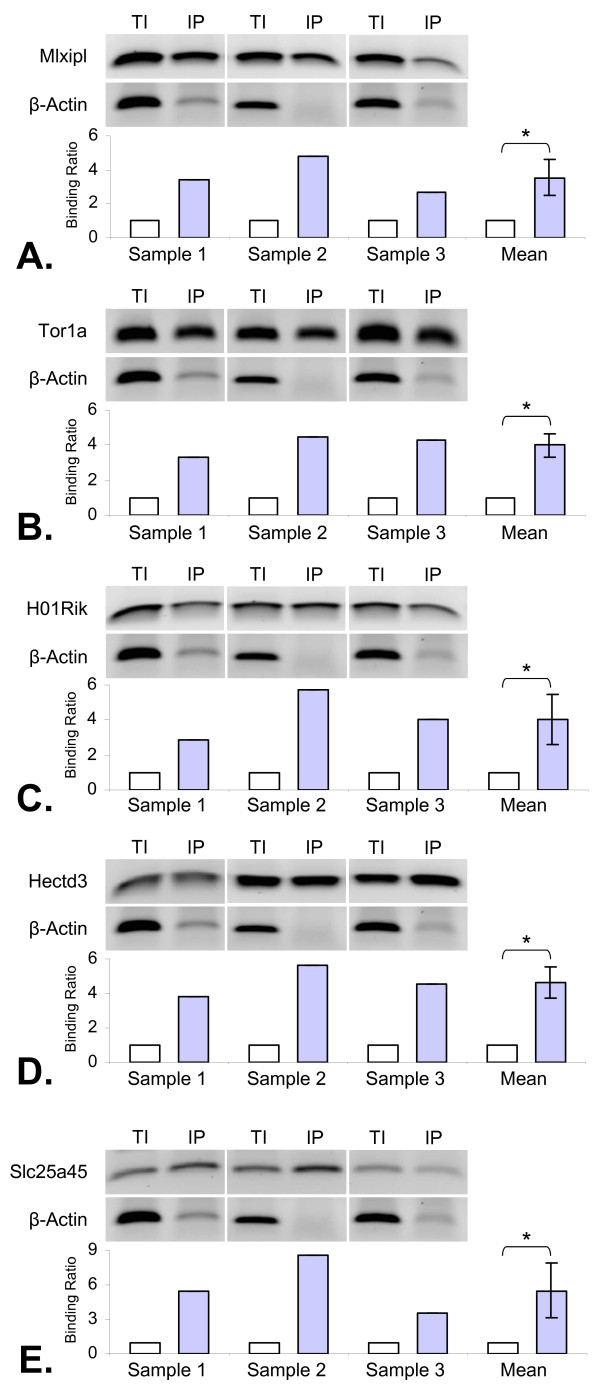
**Relative enrichment of newly identified TREs determined by ChIP-PCR**. The top half of each section shows the amplicons run on an agarose gel, and the bottom half shows relative enrichment of the immunoprecipitated (IP) samples and the total input (TI) samples when compared to β-actin enrichment. ChIP-PCR validation of negative (β-actin) and positive (Mlxipl) controls are presented in section (A). Sections (B) to (E) show enrichment of Tor1a, H01Rik (2310003H01Rik), Hectd3 and Slc25a45. Asterisks (*) denote a significant difference, p ≤ 0.05, determined by student t-test. The mean enrichments (± standard deviations) are also presented for each section. All presented immunoprecipitated enrichments were significant (p ≤ 0.05 determined by student t-test) when compared to total input, except for β-actin.

ChIP-PCR, using euthyroid livers, was performed to validate TR-TRE binding of six TREs selected from the list of genes thought to contain conserved TREs (Table 4). TRβ-1 antibody was used to precipitate the protein-DNA complexes followed by PCR analysis to compare TI (not precipitated) to IP samples. Antibody specificity has previously been demonstrated [17]. Mlxipl was used as a positive control as it binds TR in its promoter region, whereas β-actin was used as a negative control. Mean enrichment of Mlxipl compared to β-actin is shown as well as the individual enrichment of the three biological samples (Figure 3A). TREs identified in the promoter regions of Tor1a, 2310003H01Rik, Hect3d and Slc25a45 were enriched by 4-fold or more in the IP compared to TI (Figure 3B-E). Candidate TREs analysed in the promoter regions of Ihh and Arl4d showed no apparent enrichment in the IP samples (data not shown).

### Electrophoretic Mobility Shift Assays

**Figure 4:**
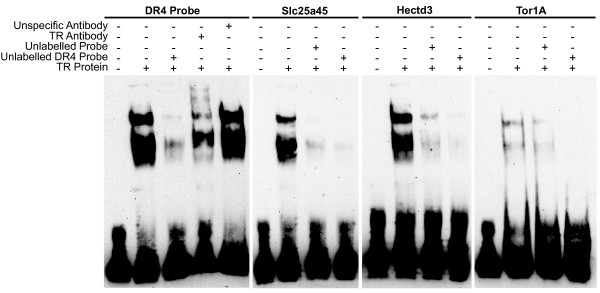
**Examining the potential binding of candidate TREs by EMSA**. The left panel shows results using a classic DR4 TRE with two "AGGTCA" half-sites. The next three panels show gel shifts when using a probe targeting candidate TREs in the promoter regions of Slc25a45, Hectd3 and Tor1a.

EMSAs were used to demonstrate the ability of the suspected TREs to bind to the TR. A DR4 containing two perfect half-sites (AGGTCA) was used as a positive control and showed a shift in electrophoretic mobility and a decrease in detection (30-fold decrease) when unlabelled probe was added (Figure 4). The DR4 positive control tested with an antibody against TRα/β exhibited a decrease in signal (7-fold decrease) of the shifted bands, and a slightly detectable supershifted band. In contrast, the non-specific mouse IgG antibody showed no reduction in detection of the shifted bands. Probes with sequences of suspected TREs associated with the genes Slc25a45, Hectd3 and Tor1a all showed a shift in electrophoretic mobility, as well as a decrease in detection when unlabelled probe (24, 17 and 2.5-fold decrease, respectively) or unlabelled DR4 positive control probe (92, 68 and 20-fold decrease respectively) was added (Figure 4). Thus, the EMSA results help to support the idea that the TR can bind to putative TREs in the promoter regions of Slc25a45, Hectd3 and Tor1a.

## Discussion

To identify direct transcriptional targets of TH and genes containing TREs, we produced global gene expression profiles in hypothyroid and hyperthyroid mouse models established by short-term exposure to MMI and perchlorate and/or TH injection. PND 15 livers were studied as animals of this age have circulating T4 levels higher than at any other age [20] and the liver is widely studied as a model of TH action. In the hypo pups TH levels were altered for three days, whereas in the hyperthyroid pups TH levels were altered for four hours immediately before tissue collection. Concentrations of anti-thyroid agents used in the hypo exposure and concentrations of injected THs were not unusually high when compared to other similar studies [17,33]. Several hundred genes were responsive to this short-term treatment, with fold-changes as high as 13.8. However, the vast majority of these genes had relatively small fold-changes (i.e., < 3-fold). When filtered on a 2.0 fold-change cutoff, only 31 genes in females and 27 genes in males were perturbed, with an overlap of 14 genes.

PCA confirmed that the microarray data clustered by sex and by treatment type suggesting that there are significant differences at the level of gene expression between i) male and female pups and ii) control, hyper, hypo and hypo+ pups. GO analysis identified multiple pathways involved in oxidative stress and xenobiotic metabolism/signalling, as well as confirming the enrichment of genes in TR/RXR activation (Additional File 3). The focus of the present research was to identify direct TH-target genes, rather than conduct a detailed mechanistic analysis. Thus, the data were mined specifically to identify direct TH-regulation and search promoters for putative TREs.

As previously mentioned, studies characterizing TH hepatic gene regulation have been carried out by others [33-36] and by our group [18]. Although these studies were carried out using different experimental designs, animal models, and developmental stages, some important similarities in molecular functions and processes emerge including genes involved in TR and RXR activation (see Additional File 3). In addition, several specific genes previously shown to be involved in TH hepatic gene regulation, such as Me1, Thrsp and Dio1 were also found in the current study. However, our study design is unique relative to the other work described above because it is a short-term transient perturbation in TH that queries a specific developmental period to identify immediately responsive genes. The overarching goal of the work is to use a robust microarray analysis of gene expression profiling coupled with a novel TRE search algorithm to identify putative TREs. Thus, a detailed functional analysis of the perturbed genes in this study compared to the other studies has not been carried out.

To identify genes under direct TH-regulation we examined the expression of a subset of responsive genes in the hypo+ and hypo++ groups in detail. These groups were rendered hypothyroid for the majority of the 3 day exposure, and then received an *i.p*. TH injection four hours before decapitation and tissue collection. In other words, only a four hour period was allowed for transcriptional response to the TH surge. Where the direction of the transcriptional fold-change in the hypo+ group was the same as in the hyper group, but the opposite of the hypo group, we predicted that the gene in question was under direct TH-regulation. Transcript levels of some of these genes were analysed by RT-qPCR in the hypo+ and hypo++ groups (Table 3). RT-qPCR analysis confirmed a significant change in the hypo+ group for four out of the nine genes relative to vehicle controls. Analysis of the hypo++ group, which registered a much higher level of circulating serum T4 compared to hypo+, showed that seven out of the nine genes had significant fold-changes relative to control. Validation of the transcriptional response in the hypo+ and hypo++ groups help to further support our assumption that the identified genes (Table 1) are under direct TH-regulation. None of the genes identified as being directly regulated by THs have characterized TREs. Although specific genes that are commonly considered to be directly regulated by TH, such as Me1, Thrsp or Dio1, were confirmed to be differentially regulated in our experiment, they did not meet the stringent requirements set for this particular evaluation (i.e., our list of potentially directly regulated genes; Table 1). As previously described, genes were considered to be directly regulated by TH if they followed a specific expression pattern. Only genes exhibiting fold-changes with FDR-adjusted p ≤ 0.05 were considered for this analysis. However, the RT-qPCR analysis demonstrated that known TH responsive genes were responding as predicted in the animal model used (Table 2).

We retrieved the relevant DNA sequences and identified potential TREs within the promoter regions of the responsive genes (i.e., the genes in Table 1). We assumed that genes under direct TH-regulation should contain functional TREs in their promoter regions. For practical reasons, our search was limited to the immediate promoter region (from 8 kb upstream to 2 kb downstream of the TSS) even though not all response elements are located in this area. Some studies have shown that transcription factor binding sites can be exceptionally far from the chromosomal location of the genes they regulate. For example, some estrogen response elements (EREs) can be found 50 kb away from of the TSS [37], whereas others can be more than 100 kb away from the TSS [38]. In addition, interchromosomal regulatory interactions have also been documented [39]. Our gene expression profiling analysis identified 28 genes that appeared to be under direct TH-regulation. Within these 28 genes, bioinformatics mining for three different types of TREs (DR4, IR0 and ER6) revealed 196 candidate TREs. To help identify true TREs, we carried out a local alignment of promoters of these mouse genes with the promoter regions of *Rattus norvegicus *and *Homo sapien*. In the -8 kb to +2 kb promoter regions of these genes we were able to identified 33 TREs in 13 different genes. Thus, within the region examined approximately 46% of the genes possessed potentially conserved TREs that fall within the established half-site criteria.

Conservation of a stretch of DNA between all three species provides evidence of the selective importance of response element functionality. Two previously characterized TREs are presented at the bottom of Table 4. The TRE near Klf9 has been characterized in mice, rats, and humans [40]. Comparison of the TREs within the orthologous Klf9 gene of the three species shows that the TREs are within 2 kb of each other relative to the TSS. The TRE located in the Mbp gene promoter region has been characterized in mice and rats [41,42]. The TREs for Mbp for both species are within 2 kb of each other relative to the TSS and show very good half-site sequence conservation. Conservation of response elements between species has been previously observed. Similar to the current study, Bourdeau *et al*. identified EREs in 660 different pairs of orthologs between mice and humans [43]. EREs identified in proximity of these orthologs were within 2 kb of their respective TSSs. Some EREs that were conserved between mice and humans were perfect matches, whereas others had multiple mismatches in their two half-site. EREs are very similar to TREs; they are both made up of AGGTCA half-sites. The classic ERE organization is an IR3, where the two half-sites are arranged in a palindromic organization separated by a 3 bp spacer. The classic TRE, on the other hand, is a DR4 with the two tandem repeated half-sites separated by a 4 bp spacer [44], although IR0, ER6 and other TREs have also been characterized. The similarities and differences that exist between EREs and TREs suggest that the underlying mechanisms that confer response element specificity are much more complicated than was once thought. A recent paper by Phan *et al*. points out some interesting findings about nuclear receptor DNA recognition specificity [45]. The authors looked at the half-site recognition for retinoic acid receptor (RAR) and TR, which both bind to the AGGTCA half-site sequences. It was once thought that the spacing between half-sites was the only aspect that conferred specificity [46-48] although the authors discovered that there were other properties that played an important role, which included naturally occurring non-consensus half-sites, flanking sequences, and auxiliary proteins produced by the cell. These additional properties make the identification of response elements even more complex, especially considering the high degree of degeneracy of TREs; nevertheless, they are important to take into consideration. It is also important to point out that our model did not consider flanking sequences and that the PWM was built with "known" TREs that biased towards the AGGTCA classic DR4 organization. We are hopeful that future response element search tools will be more flexible and take additional details such as those described above into consideration.

Previous studies have primarily used search algorithms that allow very little variation from the classic AGGTCA half-sites. Search algorithms often only allow substitution of a position towards one specific base pair, for example, position one of the half-site would only allow for an A or a G. This is a very restricted approach since TREs can be very degenerated and, based on previously characterized TREs, position one (as well as other positions) could potentially have any of the four nucleotides. Moreover, previous search algorithms often did not allow substitutions to occur at all half-site positions and often limited the number of "mismatches" when compared to the classic AGGTCA TRE half-site. Our TRE search algorithm was much more flexible when compared to these other approaches, as the PWM scored all possible TREs and applied a score based on the probability for a given base to be located at any position of the half-site based on known functional TREs. A cut-off value was applied to this criterion to minimize false-positives. Since our approach allowed for a great deal of half-site sequence variation, a filtering or refining step was needed to reduce false-positives. We found that a cross-species comparison was an effective method to reduce the amount of false-positives, and also increases the potential utility of the new TREs for future research as they may be relevant in multiple species. Our method allowed for a larger amount of variability and as a result we identified new candidate TREs that contain some features of the classic TRE, but are substantially different.

ChIP-PCR was used to validate some of the TREs identified by the bioinformatics search. Six candidate TREs were considered for this validation work. Four showed enrichment by TR-immunoprecipitation, whereas the two others did not show enrichment. This suggests that the FDR is approximately 33%, but our sample size for this estimate is quite low. The lack of identification of TREs in certain genes could be attributed to various factors. TREs could be present in the promoter regions of genes thought to be directly regulated by TH but are undetected by the bioinformatics tools currently available (i.e., do not exhibit the classic characteristics). Functional TREs have been described with a high level of degeneration, multiple spacer sizes and various half-site organizations [18,49-52] with a structure divergent from the models on which our identification algorithm was based. Consequently our model may have been too conservative to identify other functional TREs in these genes. Alternatively, TREs may truly be absent from the promoter regions of these genes. Expression may be tied to TH action through intermediate regulatory mechanisms. Non-genomic actions of TH have also been characterized [53], by which TH activation of plasma membrane receptors induces signal transduction pathways leading to various genomic or cellular responses, although some claim that the non-genomic effects of THs do not play a significant role during vertebrate development [54]. MicroRNAs (miRNAs) could also potentially regulate TH-mediated mRNA expression. We recently identified significant alterations in 40 different miRNAs in the livers of PND 15 hypothyroid mice [32]. Lastly, within our list of significantly altered genes (FDR adjusted p ≤ 0.05), several transcription factors are present, including Rarβ, Esr1 and Nr3c1 (see Additional File 1 and 2). Thus, genes present within our list may be under the control of other transcription factors that are directly regulated by THs. For example, Esr1 expression is decreased in hypothyroid female mice. This could lead to many downstream transcriptional effects since Esr1 is a transcription factor that has been shown to interact with 18 different nuclear receptors (Nuclear Receptor Signaling Atlas, http://www.nursa.org).

ChIP-PCR revealed significant enrichment for some of the stretches of DNA thought to be involved in TR interactions. TRs are thought to be bound in both presence and absence of THs, thus ChIP-PCR was only performed with euthyroid animals. Importantly, the PCR primers targeted a DNA fragment with an average size of 168 bp which includes the putative TRE. In total, four TRE sites were validated by ChIP-PCR. The TREs identified in the promoter regions of Tor1a and Slc25a45 correspond to a DR4 organization. The TRE identified in the promoter region of Hectd3 corresponds to an ER6 organization, whereas the TRE identified in the promoter region of 2310003H01Rik corresponds to an IR0 organization. The genomic locations of these newly identified TREs all fall in upstream promoter regions, -2.3, -4.6, -7.4 and -2.3 kb from their respective TSS. Most TREs identified to date in the mouse genome are located in upstream promoter regions, and more then 70% are within the 0 to -2.5 kb upstream region. The TRE that is the furthest from a TSS that has been characterized to date in mice is in the promoter region Klf9, and is -3.8 kb from its TSS. This information is based on the 14 mouse TREs that we were able to find in the literature (list available on request). Since 50% of the TREs we have characterized here are outside these bounds, our findings suggest that TREs are not necessarily found more often within the 2 or 3 kb upstream promoter region. The TRE associated with 2310003H01Rik was not validated by EMSA. This could be due to the fact that recombinant purified protein samples were used, as opposed to tissue or cellular extracts. Extracts would include other transcription factors, such as TR's known heterodimer partner RXR, which may be required for the DNA-protein interaction to occur at a detectable level. In contrast, the TREs associated with Slc25a45, Hectd3 and Tor1a were validated by EMSA, each showing a shift in the presence of TR proteins and a decrease in detection when unlabelled probe or DR4 unlabelled probe was added. The addition of unlabelled probe demonstrates the specificity of the shifted band, whereas the addition of the unlabelled DR4 probe demonstrates that the shifted band was caused by an interaction with the TR protein, since a supershift using an antibody against TR was shown for the DR4 positive control probe. A correlation between the ChIP-PCR and EMSA results was observed. Scl25a45 has the strongest enrichment measured by ChIP-PCR, followed by Hectd3 and Tor1a. Slc25a45 again showed the strongest decrease in signal intensity when unlabelled specific probe was added in the EMSA, followed by Hectd3 and Tor1a.

Slc25a45 is involved in the transportation of molecules across the mitochondrial membrane [55]. THs have been shown to induce mitochondrial biogenesis and enhance ATP production [56]. The identification of a TRE in the vicinity of the TSS of this transporter could be an indication of its involvement in TH-dependant mitochondrial biogenesis. Hectd3 is a ubiquitin ligase. In humans, it has been shown to directly bind TARA, a guanine nucleotide exchange factor involved in regulating actin cytoskeletal reorganization, cell mobility, and cell growth [57]. THs have also been shown to play a role in cytoskeletal protein regulation [58]. Thus, a TRE in the promoter region of Hectd3 could link this gene to cytoskeletal regulation via THs. In mice 2310003H01Rik is the predicted ortholog of FAAP100, Fanconi anemia-associated protein, 100 kDa [59]. FAAP100 is involved in the Fanconi anemia (FA) core complex, which plays a role in the DNA damage response network [59]. FA is a genetic disease that results from defects in proteins involved in DNA repair. People affected by FA have physical anomalies, short stature, and are predisposed to cancer arising from chromosomal instability [60]. Children with FA have a high risk of endocrine abnormalities including hypothyroidism [61]. A recent study found that TH therapy could improve the linear growth of children suffering from FA [60]. The identification of a TRE in the promoter region of 2310003H01Rik could shed light on the underlying mechanisms leading to hypothyroidism in FA patients. Tor1a is an adenosine triphosphatase. Mutation of this gene has been linked to early onset dystonia and Parkinsonism [62]. Dystonia is a disorder characterized by sustained muscle contractions often causing twitching and repetitive movements or abnormal posture. THs are important for proper muscle development and maintenance [63]. Thus, the presence of a TRE in the promoter region of Tor1a could suggest a potential role for TH-Tor1a interaction in muscle development.

## Conclusions

Using transcriptional profiling analysis we were been able to identify genes suspected of being directly regulated by THs in male and female mice. Bioinformatics mining of the promoter regions of these genes revealed 33 candidate mouse TREs potentially conserved between rats and humans. ChIP-PCR and EMSAs were employed to validate the mouse TREs. We provide evidence of four new TREs in the promoter regions of Tor1a, Slc25a45, Hectd3 and 2310003H01Rik. The results provide data to develop a stronger model for the TRE sequence motifs that direct TR binding, identify key genes that may be important for TH mediated effects, and help to further characterize the mechanisms by which TH directly regulate gene expression.

## Authors' contributions

Conceived and designed the experiments: MP, HD, MW and CY. Performed the experiments: MP, HD and RG. Analyzed the data: MP, RG, AW and MM. MP drafted the manuscript. All authors revised and approved the final manuscript.

## Supplementary Material

Additional file 1 **Altered genes in male pups, with a FDR-adjusted p < 0.05 in at least one of the treatment conditions**. Click here for file

Additional file 2 **Altered genes in female pups, with a FDR-adjusted p < 0.05 in at least one of the treatment conditions**. Click here for file

Additional file 3 **The five most affected pathways, using the list of all significant genes (significant in at least one treatment condition), along with the names of the genes that were affected by TH treatment within the pathway**. Click here for file
